# Synthesis, crystal structure and Hirshfeld surface analysis of 5,5-diphenyl-3-(prop-2-yn-1-yl)imidazolidine-2,4-dione

**DOI:** 10.1107/S2056989025003391

**Published:** 2025-04-24

**Authors:** Abderrazzak El Moutaouakil Ala Allah, Chiara Massera, Walid Guerrab, Abdulsalam Alsubari, Joel T. Mague, Youssef Ramli

**Affiliations:** a Laboratory of Medicinal Chemistry Drug Sciences Research Center Faculty of Medicine and Pharmacy Mohammed V University in Rabat Morocco; b Dipartimento di Scienze Chimiche, della Vita e della Sostenibilità Ambientale, Università di Parma, Parco Area delle Scienze 17/A 43124 Parma, Italy; c Laboratory of Medicinal Chemistry, Faculty of Clinical Pharmacy, 21 September University, Yemen; d Department of Chemistry, Tulane University, New Orleans, LA 70118, USA; Katholieke Universiteit Leuven, Belgium

**Keywords:** crystal structure, hydantoin, alkyl­ation, Hirshfeld surface analysis

## Abstract

The new phenytoin analogue 5,5-diphenyl-3-(2-propyn-1-yl)imidazolidine-2,4-dione, C_18_H_14_N_2_O_2_ (**3**), was obtained through an alkyl­ation reaction with propargyl bromide *via* the phase-transfer catalysis method, and its structure was determined *via* single-crystal X-ray diffraction analysis. The asymmetric unit of **3** consists of two independent mol­ecules differing mainly in the orientation of the propynyl group. Each mol­ecule forms an inversion dimer through pairs of N2—H2⋯O2 hydrogen bonds. The crystal structure is further consolidated by C—H⋯O and C—H⋯π inter­actions. The contributions of the different inter­actions towards the crystal packing were further analysed using Hirshfeld surface and fingerprint plots, showing that the largest contribution comes from the H⋯H contacts (45%).

## Abstract 2

The mol­ecular structure and crystal packing of 5,5-diphenyl-3-(2-propyn-1-yl)imidazolidine-2,4-dione were studied using single-crystal X-ray diffraction and Hirshfeld surface analysis..

## 1. Chemical context

Hydantoin, also known as glycolylurea or 2,4-imidazolidinedione, is a saturated heterocyclic compound derived from imidazole. Phenytoin, 5,5-di­phenyl­imidazolidine-2,4-dione, is a mol­ecule belonging to the hydantoin group, which is used in pharmacy mainly as an anti­epileptic (Giunchi *et al.*, 2019 ▸; El Moutaouakil Ala Allah *et al.*, 2024*a* ▸). The main site of action appears to be the motor cortex, where it inhibits the spread of seizure activity. Phenytoin is indicated for the control of grand mal and psychomotor seizures (Guerrab *et al.*, 2022*a* ▸). It is also applicable for various diseases, as it has anti­arrhythmic (Handzlik *et al.*, 2012 ▸), anti-HIV (Vamecq *et al.*, 1998 ▸), cytotoxic (Guerrab *et al.*, 2023*a* ▸), anti­proliferative (Aboeldahab *et al.*, 2018 ▸) and anti­bacterial effects (El Moutaouakil Ala Allah *et al.*, 2024*b* ▸). Various methods for synthesizing hydantoins have been reported, including the reaction of benzyls with urea in an ethano­lic solution of potassium or sodium hydroxide (Guerrab *et al.*, 2022*b* ▸, 2023*b* ▸; Allah *et al.*, 2024 ▸; El Moutaouakil Ala Allah *et al.*, 2023 ▸). Moreover, alkyl­ation-based chemical modifications of phenytoin are seen to strengthen and expand its biological activity (Guerrab *et al.*, 2020*a* ▸). Some analogs have also been synthesized and evaluated for their industrial properties (*e.g.* Ettahiri *et al.*, 2024 ▸). Our inter­est in hydantoins results from their simple synthesis and the ease with which X-ray quality crystals can be grown. In this context, we present in this study a new phenytoin obtained through an alkyl­ation reaction with propargyl bromide *via* the phase-transfer catalysis method. This paper presents the crystal structure of novel phenytoin analogue **3**. A Hirshfeld surface analysis was performed to analyze the inter­molecular inter­actions.
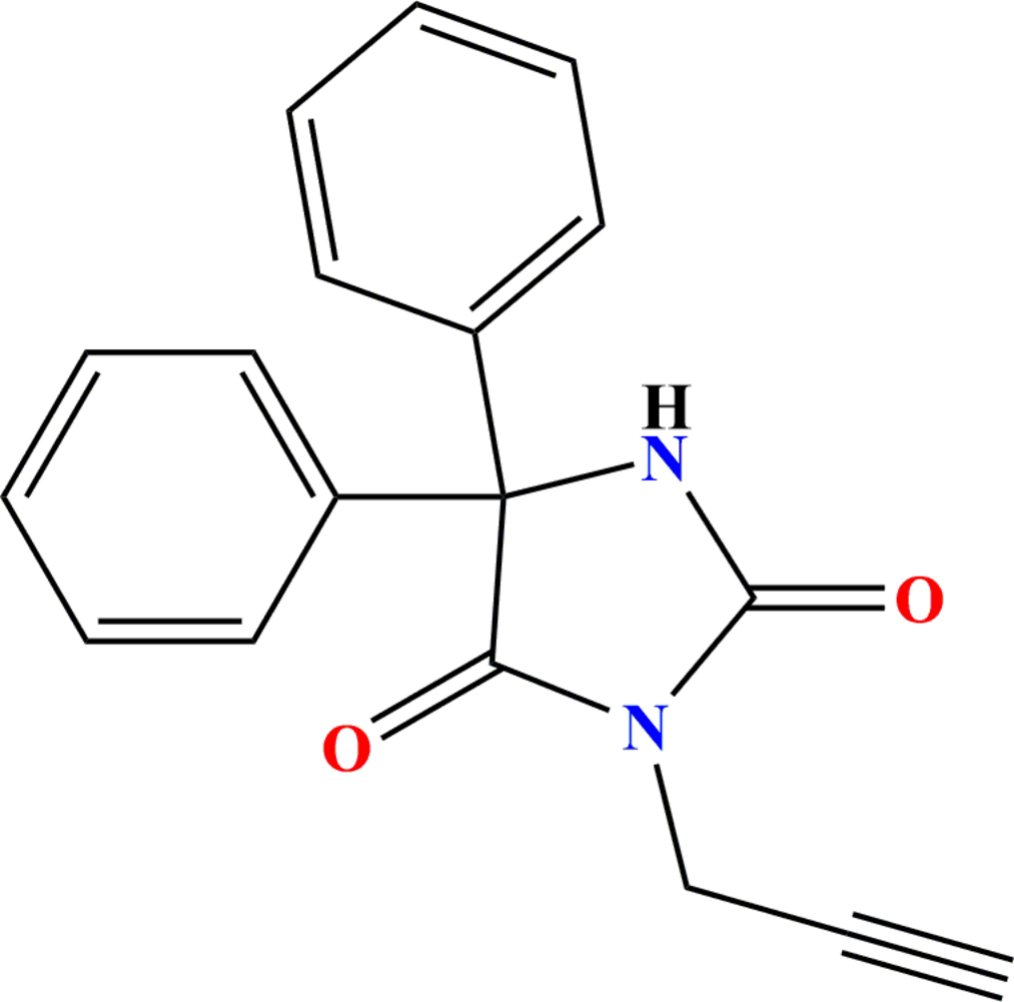


## 2. Structural commentary

**Figure 1:**
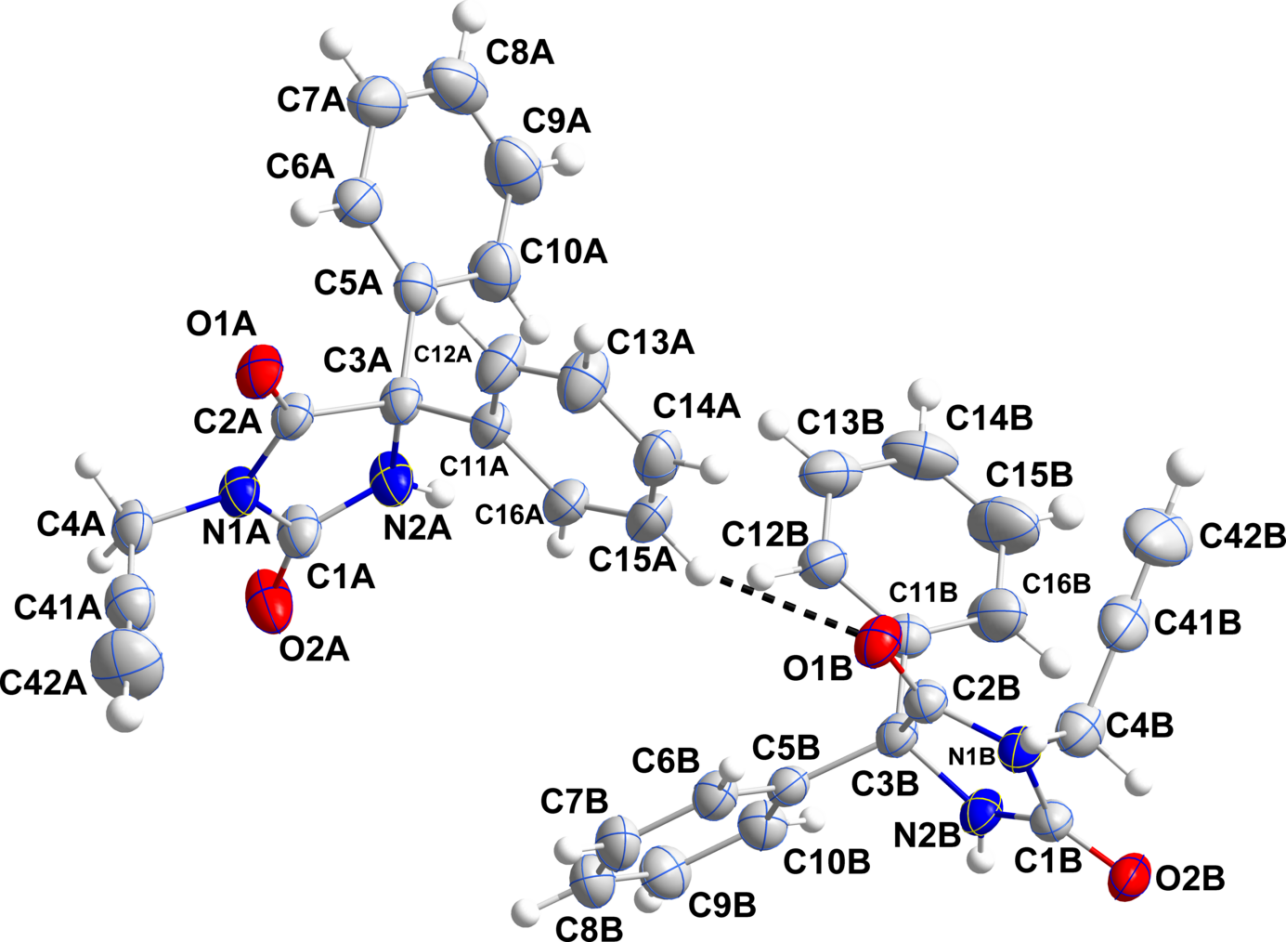
The asymmetric unit with labeling scheme and 50% probability ellipsoids. The C—H⋯O hydrogen bond is depicted by a dashed line.

**Figure 2:**
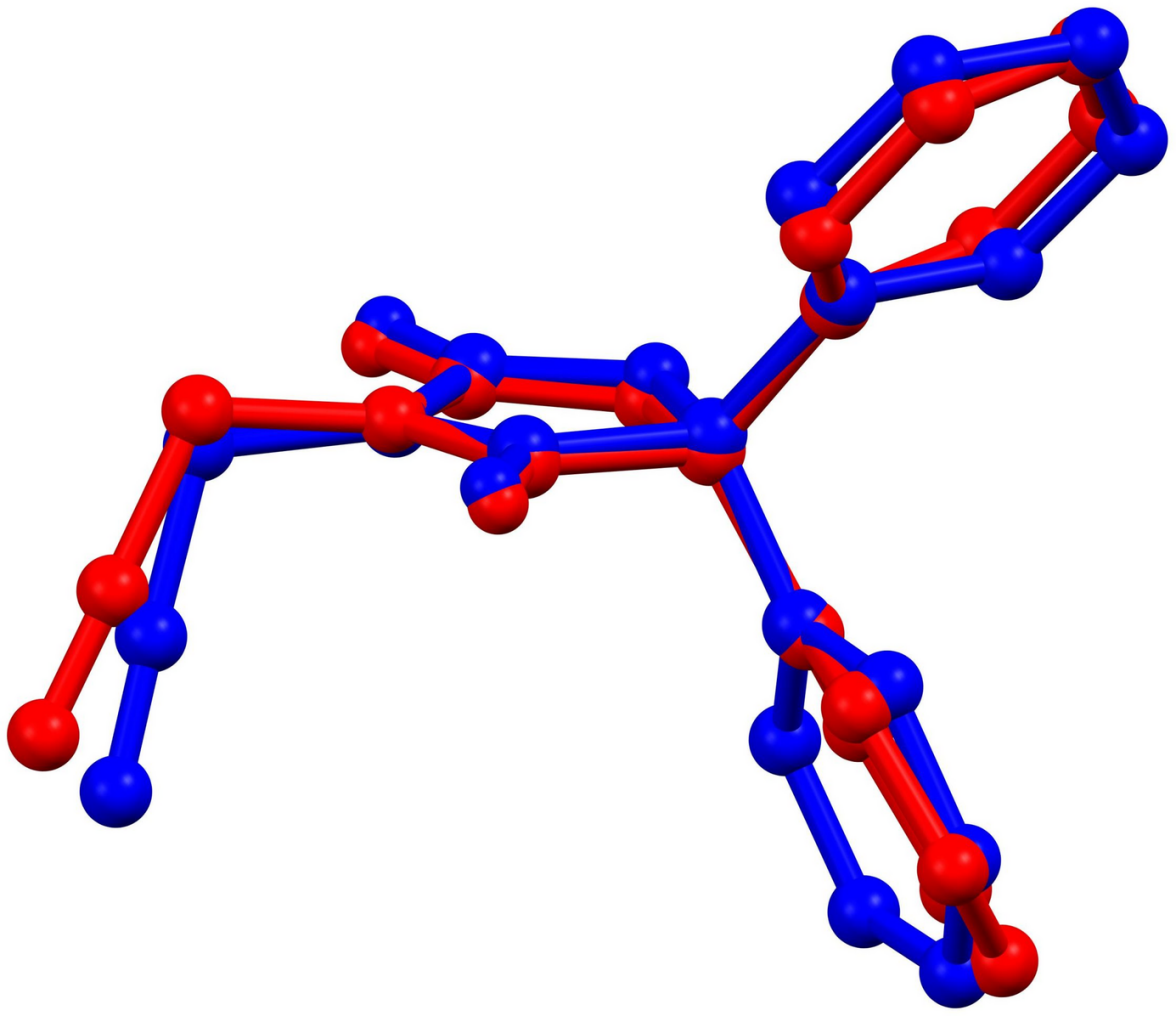
Overlay of mol­ecules *A* (red) and *B* (blue) present in the asymmetric unit of the title compound.

The asymmetric unit consists of two independent mol­ecules (*A* and *B*) differing modestly in the rotational orientations of the phenyl rings and most obviously in the orientation of the propynyl group (Fig. 1 ▸). Thus the C2*A*—N1*A*—C4*A*—C41*A* torsion angle is −80.1 (2)°, while C2*B*—N1*B*—C4*B*—C41*B* is −68.4 (2)°. The overlay of mol­ecule *A* (red) and mol­ecule *B* (blue) is shown in Fig. 2 ▸; the r.m.s. deviation for non-H atoms is 0.325 Å. As in many related mol­ecules, the dihedral angles between the mean planes of the five-membered ring and those of the phenyl rings is larger than 50°. In mol­ecule *A*, these are 53.58 (8) and 56.68 (9)° while in mol­ecule *B*, they are 56.81 (8) and 74.26 (9)°, another indication of the different conformations of the two independent mol­ecules. Bond lengths and inter­bond angles are as expected for this type of compound. The five-membered rings C1*A*–C3*A*/N1*A*/N2*A* (ring *A*) and C1*B*–C3*B*/N1*B/*N2*B* (ring *B*) are both essentially planar, with r.m.s. deviations of 0.010 and 0.044 and Å, respectively. For ring *A*, atom N1*A* shows the largest deviation [0.009 (1) Å], while atoms O1*A* and O2*A* deviate by −0.035 (1) and 0.028 (1) Å from the mean plane. For ring *B*, the largest deviation of −0.038 (2) Å is shown by atom C2*B*, while atoms O1*B* and O2*B* deviate −0.097 (1) and −0.047 (1) Å from the mean plane.

## 3. Supra­molecular features

**Figure 3:**
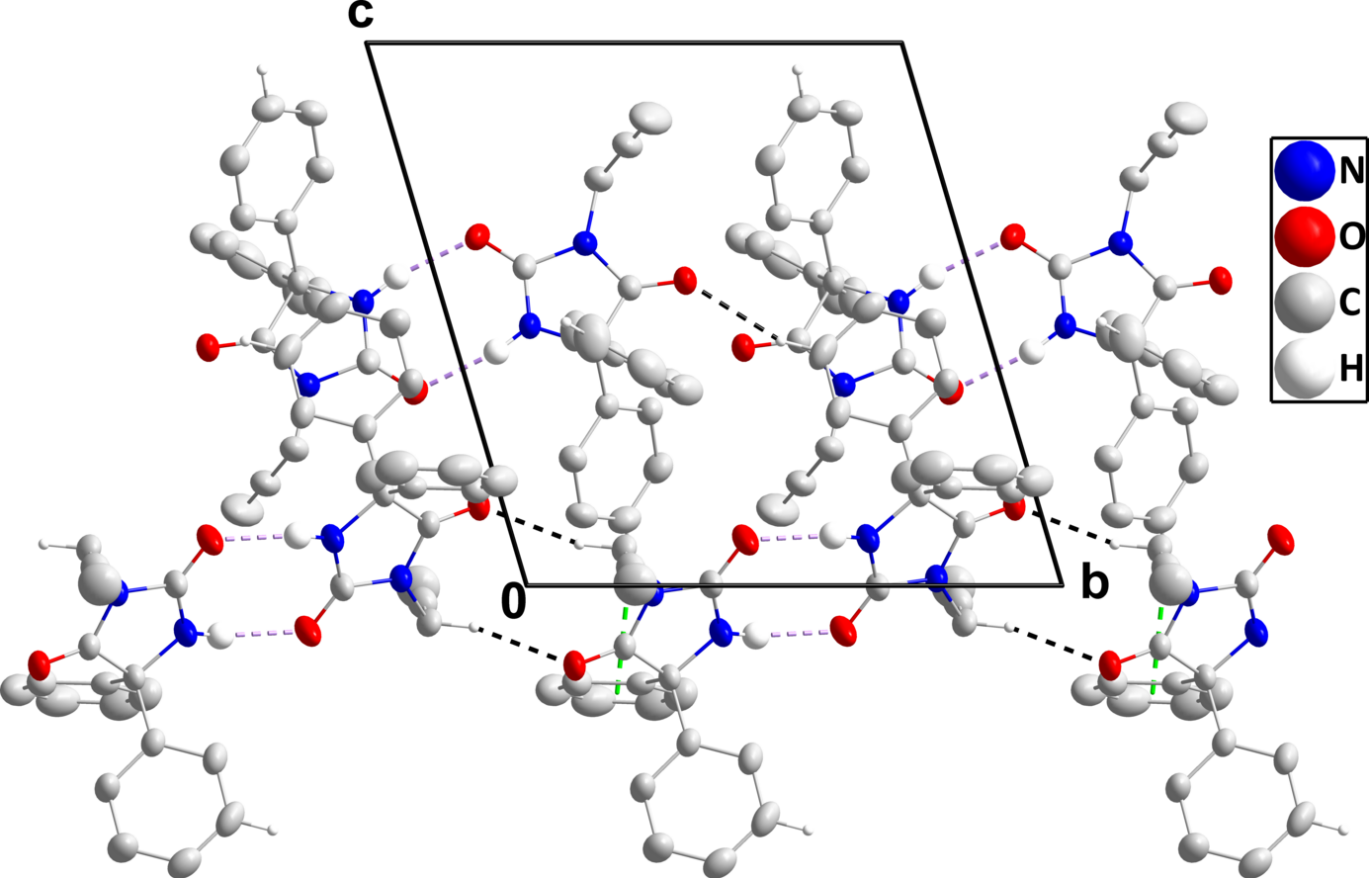
Packing viewed along the *a*-axis direction with N—H⋯O and C—H⋯O hydrogen bonds depicted, respectively, by violet and black dashed lines. The C—H⋯π(ring) inter­actions are depicted by green dashed lines and non-inter­acting hydrogen atoms are omitted for clarity.

In the crystal, each independent mol­ecule forms an inversion dimer through pairs of N2—H2⋯O2 (*A* or *B*) hydrogen bonds (Table 1 ▸). For mol­ecule *A*, these dimers are connected into chains extending along the *b*-axis direction by inversion-related C4*A*—H4*AA*⋯O1*A* hydrogen bonds (Table 1 ▸ and Fig. 3 ▸). The dimers of mol­ecule *B* are linked to the above-mentioned chains by C15*A*—H15*A*⋯O1*B* hydrogen bonds and C8*B*—H8*B*⋯*Cg*2 inter­actions (Table 1 ▸ and Fig. 3 ▸). These supra­molecular aggregates are in turn connected by C15*B*—H15*B*⋯*Cg*3 inter­actions (Table 1 ▸). *Cg*2 and *Cg*3 are the centroids of the C5*A*–C10*A* and C11*A*–C16*A* benzene rings, respectively.

## 4. Database survey

**Figure 4:**
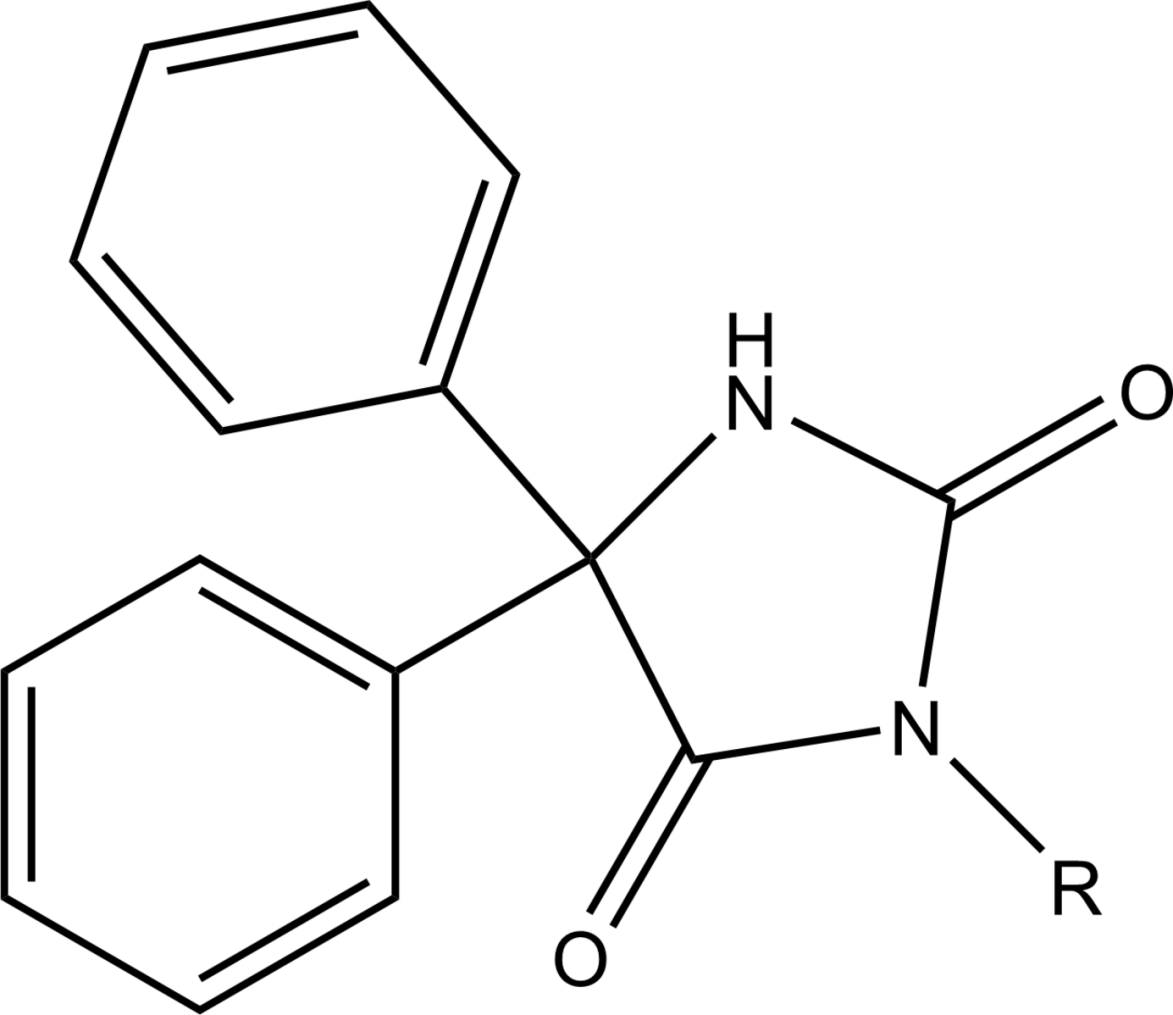
The search fragment used for the database search.

A search of the Cambridge Structural Database (CSD, 2023.3.1; Groom *et al.*, 2016 ▸) with the fragment shown in Fig. 4 ▸ (*R* = C) yielded 25 structures, of which 19 were deemed closest to the title mol­ecule, since all of the substituents, *R*, were mainly hydro­carbon groups. These are listed in Table 2 ▸ from which it is apparent that the dihedral angles between the mean planes of the two phenyl groups and that of the five-membered ring to which they are attached range from 51.23 (6)° (WUGCEJ) to as large as 83.89 (16)° (YOFMUE). The two angles may also be nearly equal as in FEHPUG or differ by as much as 31.81° as in WUGCEJ. The range of dihedral angles and the difference between them in a particular mol­ecule is likely due to packing considerations, but there does not appear to be a simple correlation with the space group or the size of the substituent *R*.

## 5. Hirshfeld surface analysis

**Figure 5:**
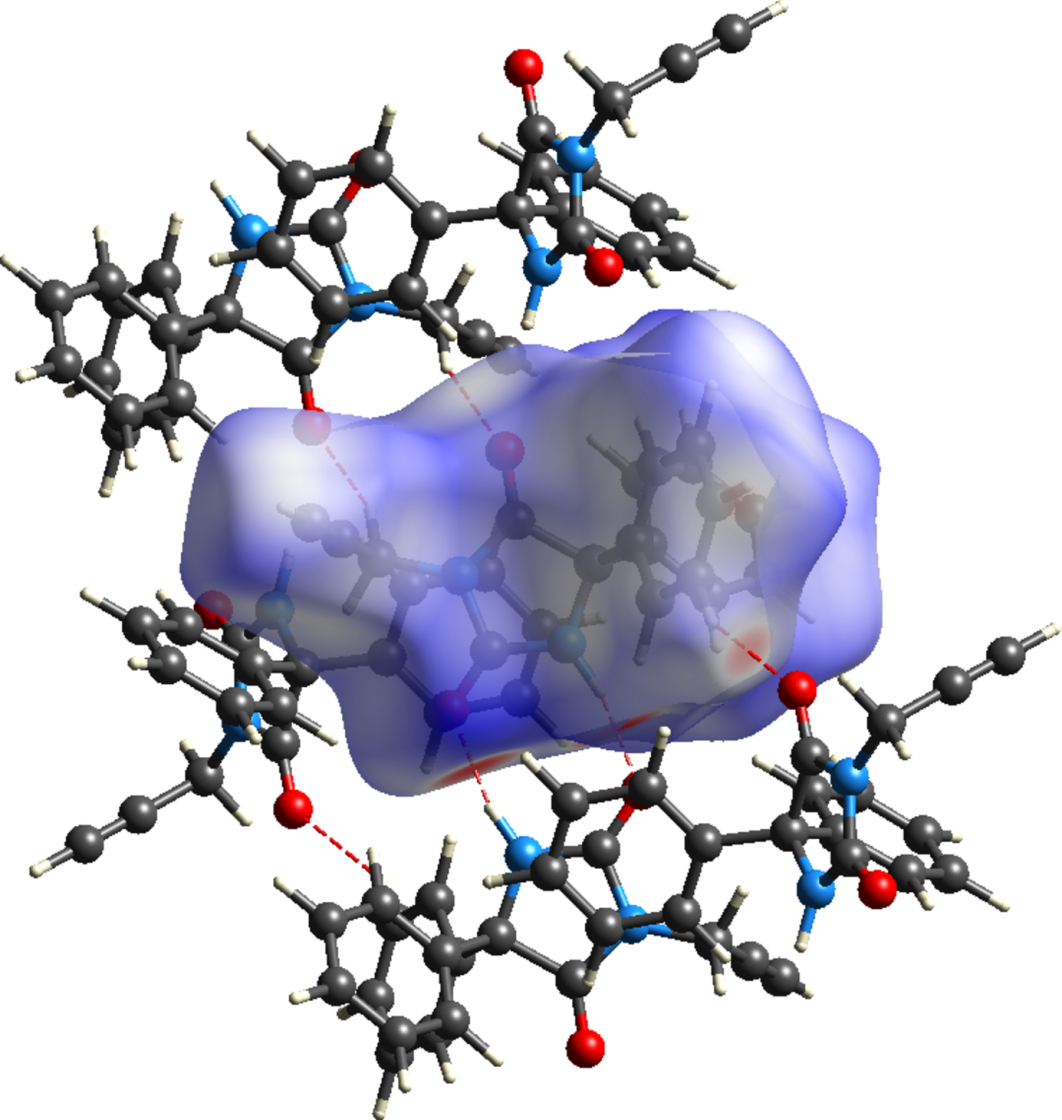
The *d*_norm_ surface for mol­ecule *A* with nearest neighbor mol­ecules *A* and *B*. The inter­molecular hydrogen bonds are depicted by red dashed lines.

**Figure 6:**
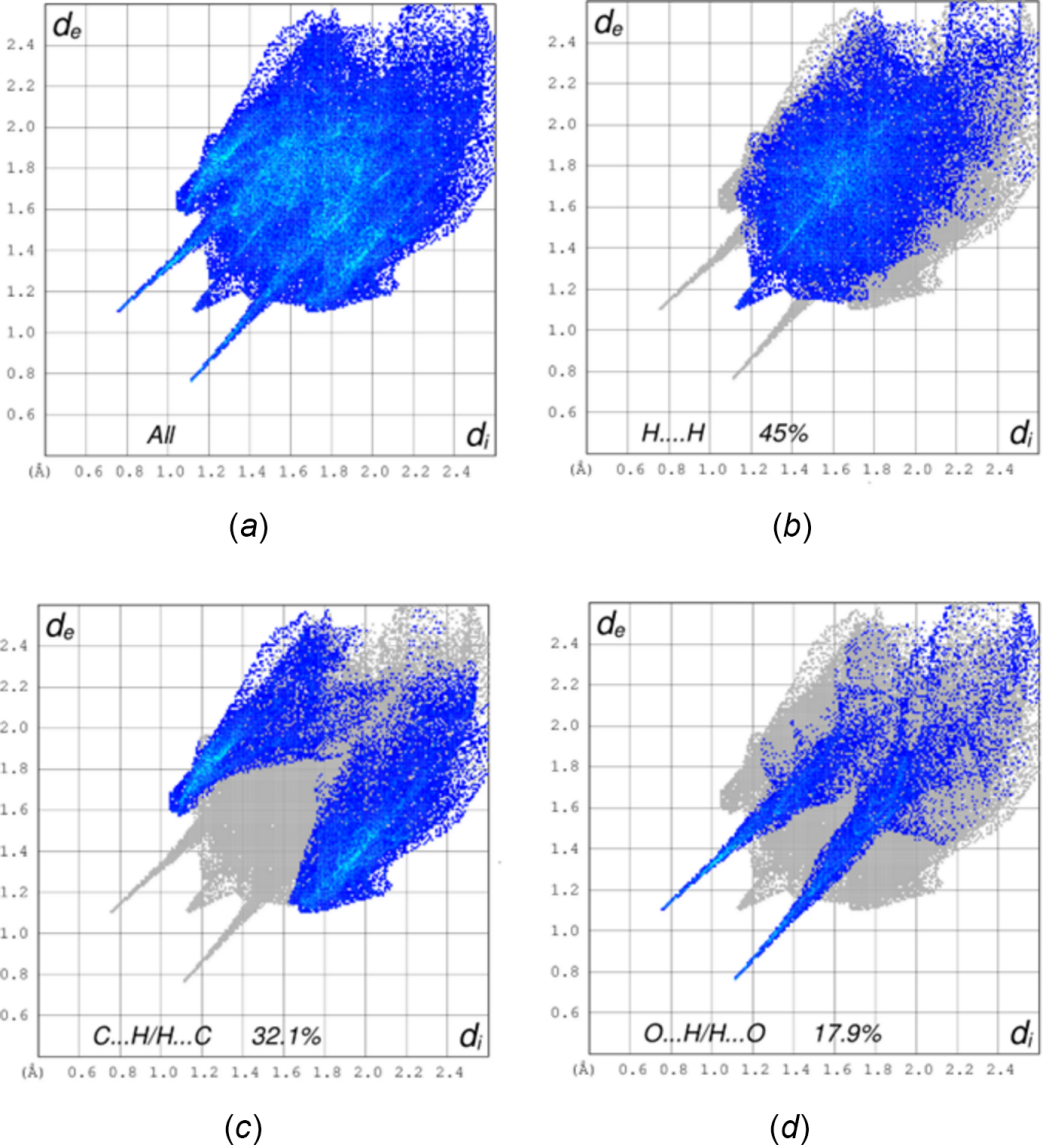
Two-dimensional fingerprint plots showing all inter­molecular inter­actions (*a*) and those showing just H⋯H contacts (*b*), C⋯H/H⋯C contacts (*c*) and O⋯H/H⋯O contacts (*d*).

*CrystalExplorer* (Spackman *et al.*, 2021 ▸) was used to perform the Hirshfeld surface (HS) analysis. A full description of the procedures and the inter­pretation of the results obtained has been published (Tan *et al.*, 2019 ▸). Fig. 5 ▸ presents the *d*_norm_ surface for mol­ecule *A*, together with several near neighbors consisting of both mol­ecules *A* and *B*. A portion of the chain of dimers formed by the *A* mol­ecules can be seen in the center of the figure, while at the bottom of the surface the N—H⋯O hydrogen bonds are shown as two intense red spots. The lighter red spot at the lower right corresponds to the C—H⋯O hydrogen bond that links mol­ecules *A* and *B*. Fig. 6 ▸ shows the 2-D fingerprint plots for all inter­molecular inter­actions (*a*) and those specifically representing H⋯H (*b*), C⋯H/H⋯C (*c*) and O⋯H/H⋯O (*d*) inter­actions. The largest contribution to the inter­molecular inter­actions comes from the H⋯H contacts (45%), which is consistent with the periphery of the mol­ecule being largely hydrogen in nature and can be attributed to van der Waals contacts. The C⋯H/H⋯C contacts contribute 32.1% and appear as a pair of blunt peaks at *d*_e_ + *d*_i_ ≃ 3.2 Å. These can be primarily attributed to the C—H⋯π(ring) inter­actions. The last significant contribution is from the O⋯H/H⋯O inter­actions (17.9%) which appear as a pair of sharp spikes at *d*_e_ + *d*_i_ ≃ 2.2 Å. These represent the N—H⋯O and the C—H⋯O hydrogen bonds, respectively. All other inter­molecular contacts, *e.g.* N⋯H/H⋯N, C⋯N, O⋯C, *etc*., contribute less than 2% to the total. The HS surface for mol­ecule *B* is virtually identical to that for mol­ecule *A* as are the 2-D fingerprint plots. The only difference is in the percentage contribution to the overall inter­molecular inter­actions. For mol­ecule *B* these are 40.3% for H⋯H contacts, 34.7% for C⋯H/H⋯C contacts and 18.8% for O⋯H/H⋯O contacts. Again, other contacts are less than 2% each.

## 6. Synthesis and crystallization

**Figure 7:**
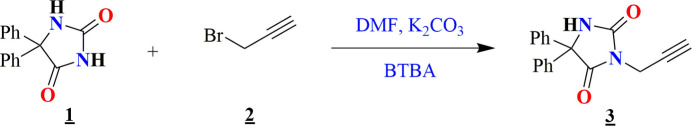
Reaction scheme for the formation of the title compound **3**.

The reaction scheme for the synthesis of the title compound is shown in Fig. 7 ▸. To a solution of phenytoin **1** (0.5 g, 2 mmol) in DMF (10 mL), in the presence of K_2_CO_3_ (2.2 mmol), propargyl bromide **2** (2.2 mmol) was added dropwise along with a catalytic amount of BTBA (benzyl tributyl ammonium bromide). The mixture was stirred at room temperature for 2 h. After filtration of the salts, the solvent was evaporated, and the resulting residue was purified by recrystallization in ethanol, yielding colorless crystals of **3**.

**Yield** = 96%, **m.p.** = 408–410 K. **FT-IR** (ATR, cm^−1^): 3375 (CH proparg­yl), 3060–3080, (CH aromatic), 1765 (C=O); **^1^H NMR** (500 MHz, DMSO-*d*_6_): δ ppm 3.22 (*t*, 1H, CH proparg­yl), 4,22 (*s*, 2H, N—CH_2_), 7.03–7.42 (*m*, 10, Ar—H), 9.78 (*s*, 1H, NH); **^13^C NMR**: 28.01 (N—CH_2_); 74.40 (CH proparg­yl); 69.71 (C—2Ph); 74.40 (C_q_ proparg­yl); 127.25, 128.00, 128.58, 140.15 (C—Ar); 154.57 (C=O); 172.73 (C=O). **HRMS (ESI)**: calculated for C_18_H_14_N_2_O_2_ [*M* + H]^+^ 291.1055; found 291.1122.

## 7. Refinement

Crystal data, data collection and structure refinement details are summarized in Table 3 ▸. The carbon-bound H atoms were placed in calculated positions and refined isotropically using the riding model, with C—H distances ranging from 0.95 to 0.99 Å and *U*_iso_(H) set to 1.2–1.5*U*_eq_(C). The H atoms H2*A* and H2*B* of the two imidazole rings were found in a difference-Fourier map and refined freely.

## Supplementary Material

Crystal structure: contains datablock(s) I. DOI: 10.1107/S2056989025003391/vm2311sup1.cif

Structure factors: contains datablock(s) I. DOI: 10.1107/S2056989025003391/vm2311Isup2.hkl

Supporting information file. DOI: 10.1107/S2056989025003391/vm2311Isup3.cml

CCDC reference: 2444171

Additional supporting information:  crystallographic information; 3D view; checkCIF report

## Figures and Tables

**Table 1:** Hydrogen-bond geometry (Å, °) *Cg*2 and *Cg*3 are the centroids of the C5*A*–C10*A* and C11*A*–C16*A* benzene rings, respectively.

*D*—H⋯*A*	*D*—H	H⋯*A*	*D*⋯*A*	*D*—H⋯*A*
N2*A*—H2*A*⋯O2*A*^i^	0.88 (2)	1.99 (2)	2.855 (2)	167 (2)
N2*B*—H2B*B*⋯O2*B*^ii^	0.89 (2)	1.99 (2)	2.847 (1)	163 (2)
C4*A*—H4*AA*⋯O1*A*^iii^	0.99	2.31	3.253 (2)	160
C15*A*—H15*A*⋯O1*B*	0.95	2.44	3.332 (2)	156
C8*B*—H8*B*⋯C*g*2^i^	0.95	2.88	3.713 (2)	147
C15*B*—H15*B*⋯C*g*3^iv^	0.95	2.87	3.742 (3)	153

Symmetry codes: (i) 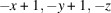; (ii) 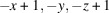; (iii) 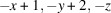; (iv) 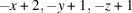.

**Table 2:** Dihedral angles (°) between the phenyl rings and the five-membered ring for related mol­ecules

*R*	Refcode	Dihedral angles	Reference
Me	PEPDUM	59.17 (6), 53.21 (6)	Guerrab *et al.* (2017*a* ▸)
Et	FEHPUG	64.03 (5), 63.04 (5)	Guerrab *et al.* (2017*b* ▸)
2-bromo­eth­yl	NIBMOE	63.60 (16), 76.45 (16)	Guerrab *et al.* (2023*a* ▸)
all­yl	BUCDEL	62.07 (13), 64.55 (12)	Guerrab *et al.* (2020*a* ▸)
*n*-prop­yl	WEMQUD	66.09 (8), 67.12 (8); 64.48 (8), 71.25 (8)	Guerrab *et al.* (2017*c* ▸)
*n*-prop­yl	WEMQUD01	64.6 (8), 69.3 (8)	Trišović *et al.* (2019 ▸)
*i*-prop­yl	YOFMOY	56.86 (11), 79.79 (11)	Trišović *et al.* (2019 ▸)
cyclo­prop­yl	YOFMUE	59.52 (15), 83.89 (16)	Trišović *et al.*, 2019 ▸)
*i*-but­yl	QENBET	50.08 (6), 66.31 (5)	Guerrab *et al.* (2018*a* ▸)
*s*-but­yl	YEDYOZ	68.42 (5), 73.04 (5)	Guerrab *et al.* (2022*b* ▸)
*t*-but­yl	YOFNAL	66.8 (2), 73.8 (2)	Trišović *et al.* (2019 ▸)
*n*-pent­yl	YOFNEP	63.41 (16), 75.12 (16)	Trišović *et al.* (2019 ▸)
*n*-hex­yl	GEMSOJ	63.6 (8), 70.4 (8)	Guerrab *et al.* (2017*d* ▸)
*n*-oct­yl	QENBOD	69.71 (12), 71.80 (12); 71.24 (11), 67.85 (12)	Guerrab *et al.* (2018*b* ▸)
*n*-non­yl	QAGPAT	76.0 (8), 63.5 (8)	Guerrab *et al.* (2020*b* ▸)
*n*-dec­yl	PAJMAS	54.03 (7), 60.67 (7)	Guerrab *et al.* (2021 ▸)
benz­yl	MESSAH	71.65 (6), 71.62 (6); 76.38 (6), 70.22 (6)	Guerrab *et al.* (2018*c* ▸)
phen­yl	WUGCEJ	51.23 (6), 83.04 (6)	Berntsen *et al.* (2020 ▸)
*m*-tol­yl	WUGCIN	67.28 (8), 65.51 (8)	Berntsen *et al.* (2020 ▸)

**Table 3:** Experimental details

Crystal data	
Chemical formula	C_18_H_14_N_2_O_2_
*M*_r_	290.31
Crystal system, space group	Triclinic, *P* 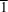
Temperature (K)	200
*a*, *b*, *c* (Å)	11.3526 (3), 12.0162 (3), 13.3087 (3)
α, β, γ (°)	97.080 (1), 114.406 (1), 107.335 (1)
*V* (Å^3^)	1513.47 (7)
*Z*	4
Radiation type	Cu *K*α
μ (mm^−1^)	0.68
Crystal size (mm)	0.17 × 0.15 × 0.10

Data collection	
Diffractometer	Bruker D8 Venture PhotonII
Absorption correction	Multi-scan (*SADABS*; Krause et al., 2015 ▸)
*T*_min_, *T*_max_	0.639, 0.754
No. of measured, independent and observed [*I* > 2σ(*I*)] reflections	17923, 5879, 5126
*R*_int_	0.040
(sin θ/λ)_max_ (Å^−1^)	0.618

Refinement	
*R*[*F*^2^ > 2σ(*F*^2^)], *wR*(*F*^2^), *S*	0.040, 0.117, 1.02
No. of reflections	5879
No. of parameters	406
H-atom treatment	H atoms treated by a mixture of independent and constrained refinement
Δρ_max_, Δρ_min_ (e Å^−3^)	0.23, −0.23

Computer programs: *APEX3* and *SAINT* (Bruker, 2016 ▸), *SHELXT2018/2* (Sheldrick, 2015*a* ▸), *SHELXL2019/2* (Sheldrick, 2015*b* ▸), *Mercury* (Macrae *et al.*, 2020 ▸) *DIAMOND* (Brandenburg & Putz, 2012 ▸), *WinGX* (Farrugia, 2012 ▸), *publCIF* (Westrip, 2010 ▸) and *enCIFer* (Allen *et al.*, 2004 ▸).

## supplementary crystallographic information

### 5,5-Diphenyl-3-(2-propyn-1-yl)imidazolidine-2,4-dione Crystal data

**Table:**

C_18_H_14_N_2_O_2_	*Z* = 4
*M**_r_* = 290.31	*F*(000) = 608
Triclinic, *P*1	*D*_x_ = 1.274 Mg m^−^^3^
*a* = 11.3526 (3) Å	Cu *K*α radiation, λ = 1.54178 Å
*b* = 12.0162 (3) Å	Cell parameters from 1278 reflections
*c* = 13.3087 (3) Å	θ = 3.8–72.3°
α = 97.080 (1)°	µ = 0.68 mm^−^^1^
β = 114.406 (1)°	*T* = 200 K
γ = 107.335 (1)°	Prismatic, colourless
*V* = 1513.47 (7) Å^3^	0.17 × 0.15 × 0.10 mm

### 5,5-Diphenyl-3-(2-propyn-1-yl)imidazolidine-2,4-dione Data collection

**Table:**

Bruker D8 Venture PhotonII diffractometer	5879 independent reflections
Radiation source: fine-focus sealed tube	5126 reflections with *I* > 2σ(*I*)
Graphite monochromator	*R*_int_ = 0.040
phi & ω scan	θ_max_ = 72.3°, θ_min_ = 3.8°
Absorption correction: multi-scan (SADABS; Krause et al., 2015)	*h* = −13→13
*T*_min_ = 0.639, *T*_max_ = 0.754	*k* = −14→14
17923 measured reflections	*l* = −16→16

### 5,5-Diphenyl-3-(2-propyn-1-yl)imidazolidine-2,4-dione Refinement

**Table:**

Refinement on *F*^2^	Hydrogen site location: mixed
Least-squares matrix: full	H atoms treated by a mixture of independent and constrained refinement
*R*[*F*^2^ > 2σ(*F*^2^)] = 0.040	*w* = 1/[σ^2^(*F*_o_^2^) + (0.0647*P*)^2^ + 0.2418*P*] where *P* = (*F*_o_^2^ + 2*F*_c_^2^)/3
*wR*(*F*^2^) = 0.117	(Δ/σ)_max_ < 0.001
*S* = 1.02	Δρ_max_ = 0.23 e Å^−^^3^
5879 reflections	Δρ_min_ = −0.23 e Å^−^^3^
406 parameters	Extinction correction: SHELXL-2019/2 (Sheldrick 2015*b*), Fc^*^=kFc[1+0.001xFc^2^λ^3^/sin(2θ)]^-1/4^
0 restraints	Extinction coefficient: 0.0056 (5)

### 5,5-Diphenyl-3-(2-propyn-1-yl)imidazolidine-2,4-dione Special details

**Table:**

Geometry. All esds (except the esd in the dihedral angle between two l.s. planes) are estimated using the full covariance matrix. The cell esds are taken into account individually in the estimation of esds in distances, angles and torsion angles; correlations between esds in cell parameters are only used when they are defined by crystal symmetry. An approximate (isotropic) treatment of cell esds is used for estimating esds involving l.s. planes.

### 5,5-Diphenyl-3-(2-propyn-1-yl)imidazolidine-2,4-dione Fractional atomic coordinates and isotropic or equivalent isotropic displacement parameters (Å^2^)

**Table:**

	*x*	*y*	*z*	*U*_iso_*/*U*_eq_	
O1A	0.59193 (10)	0.95473 (8)	0.14741 (8)	0.0443 (2)	
O2A	0.38335 (12)	0.56618 (9)	−0.08410 (9)	0.0588 (3)	
N1A	0.46487 (12)	0.76899 (9)	0.01292 (9)	0.0390 (2)	
N2A	0.59068 (12)	0.66309 (10)	0.08644 (10)	0.0436 (3)	
C1A	0.47235 (15)	0.65472 (11)	−0.00263 (12)	0.0426 (3)	
C2A	0.57528 (13)	0.85052 (10)	0.11236 (11)	0.0361 (3)	
C3A	0.67094 (13)	0.78384 (10)	0.16927 (10)	0.0360 (3)	
C4A	0.35230 (15)	0.79744 (12)	−0.06815 (11)	0.0424 (3)	
H4AA	0.392791	0.878766	−0.076517	0.051*	
H4AB	0.306232	0.737385	−0.144406	0.051*	
C41A	0.24764 (17)	0.79629 (13)	−0.03154 (13)	0.0513 (4)	
C42A	0.1633 (2)	0.7962 (2)	−0.0025 (2)	0.0825 (6)	
H42A	0.094924	0.796197	0.021040	0.099*	
C5A	0.81762 (14)	0.84068 (12)	0.18064 (10)	0.0391 (3)	
C6A	0.87396 (15)	0.95894 (13)	0.17795 (12)	0.0481 (3)	
H6A	0.819765	1.007611	0.166135	0.058*	
C7A	1.00935 (18)	1.00670 (17)	0.19244 (15)	0.0630 (4)	
H7A	1.046191	1.087114	0.188360	0.076*	
C8A	1.09019 (18)	0.9387 (2)	0.21258 (15)	0.0672 (5)	
H8A	1.183203	0.972144	0.223631	0.081*	
C9A	1.03585 (19)	0.8221 (2)	0.21666 (16)	0.0667 (5)	
H9A	1.091751	0.774849	0.230654	0.080*	
C10A	0.90012 (17)	0.77223 (16)	0.20059 (14)	0.0543 (4)	
H10A	0.863499	0.691147	0.203234	0.065*	
C11A	0.68600 (13)	0.78422 (11)	0.28908 (11)	0.0366 (3)	
C12A	0.74629 (18)	0.89481 (13)	0.37237 (12)	0.0505 (3)	
H12A	0.773528	0.968502	0.353196	0.061*	
C13A	0.7670 (2)	0.89859 (15)	0.48275 (13)	0.0564 (4)	
H13A	0.807925	0.974694	0.538940	0.068*	
C14A	0.72841 (16)	0.79222 (15)	0.51154 (13)	0.0516 (4)	
H14A	0.742334	0.794838	0.587398	0.062*	
C15A	0.66983 (16)	0.68256 (15)	0.43028 (14)	0.0528 (4)	
H15A	0.643701	0.609288	0.450356	0.063*	
C16A	0.64825 (14)	0.67748 (12)	0.31873 (13)	0.0443 (3)	
H16A	0.607769	0.601082	0.263057	0.053*	
O1B	0.67935 (10)	0.46416 (8)	0.56167 (8)	0.0430 (2)	
O2B	0.58550 (10)	0.10049 (8)	0.64236 (8)	0.0450 (2)	
N1B	0.65081 (11)	0.29799 (9)	0.63038 (9)	0.0367 (2)	
N2B	0.58273 (12)	0.14933 (9)	0.47865 (9)	0.0370 (2)	
C1B	0.60271 (12)	0.17176 (11)	0.58711 (10)	0.0357 (3)	
C2B	0.65096 (13)	0.35657 (10)	0.54846 (10)	0.0342 (3)	
C3B	0.61432 (13)	0.25927 (10)	0.44140 (10)	0.0335 (3)	
C4B	0.69356 (16)	0.35873 (13)	0.74856 (11)	0.0461 (3)	
H4BA	0.641547	0.411788	0.748697	0.055*	
H4BB	0.669560	0.297129	0.787415	0.055*	
C41B	0.84330 (18)	0.43143 (14)	0.81171 (12)	0.0557 (4)	
C42B	0.9646 (2)	0.4894 (2)	0.86070 (17)	0.0882 (7)	
H42B	1.062699	0.536214	0.900302	0.106*	
C5B	0.48929 (12)	0.24919 (10)	0.33036 (10)	0.0330 (3)	
C6B	0.41792 (14)	0.32636 (11)	0.32142 (11)	0.0393 (3)	
H6B	0.446357	0.390018	0.386763	0.047*	
C7B	0.30456 (15)	0.31056 (13)	0.21666 (13)	0.0473 (3)	
H7B	0.255485	0.363157	0.211075	0.057*	
C8B	0.26317 (15)	0.21903 (14)	0.12106 (12)	0.0488 (3)	
H8B	0.186476	0.209149	0.049625	0.059*	
C9B	0.33373 (16)	0.14167 (13)	0.12955 (12)	0.0477 (3)	
H9B	0.305049	0.078260	0.063919	0.057*	
C10B	0.44609 (14)	0.15641 (12)	0.23347 (11)	0.0405 (3)	
H10B	0.494054	0.102919	0.238765	0.049*	
C11B	0.74665 (13)	0.28834 (11)	0.42686 (10)	0.0369 (3)	
C12B	0.77757 (15)	0.37651 (13)	0.37318 (12)	0.0454 (3)	
H12B	0.716546	0.417811	0.345115	0.054*	
C13B	0.89756 (17)	0.40443 (15)	0.36044 (14)	0.0567 (4)	
H13B	0.918590	0.465199	0.324107	0.068*	
C14B	0.98625 (18)	0.34451 (17)	0.40019 (17)	0.0660 (5)	
H14B	1.066874	0.362105	0.389500	0.079*	
C15B	0.9570 (2)	0.25872 (19)	0.4557 (2)	0.0767 (6)	
H15B	1.018882	0.218367	0.484605	0.092*	
C16B	0.83838 (17)	0.23106 (15)	0.46947 (17)	0.0584 (4)	
H16B	0.819719	0.172392	0.508422	0.070*	
H2A	0.613 (2)	0.5994 (18)	0.0919 (16)	0.062 (5)*	
H2B	0.5382 (19)	0.0745 (17)	0.4319 (15)	0.052 (4)*	

### 5,5-Diphenyl-3-(2-propyn-1-yl)imidazolidine-2,4-dione Atomic displacement parameters (Å^2^)

**Table:**

	*U*^11^	*U*^22^	*U*^33^	*U*^12^	*U*^13^	*U*^23^
O1A	0.0576 (6)	0.0257 (4)	0.0468 (5)	0.0196 (4)	0.0199 (4)	0.0109 (4)
O2A	0.0652 (7)	0.0305 (5)	0.0486 (6)	0.0210 (5)	−0.0007 (5)	0.0020 (4)
N1A	0.0456 (6)	0.0270 (5)	0.0393 (5)	0.0178 (4)	0.0128 (5)	0.0107 (4)
N2A	0.0508 (6)	0.0253 (5)	0.0426 (6)	0.0194 (5)	0.0092 (5)	0.0058 (4)
C1A	0.0514 (7)	0.0281 (6)	0.0403 (7)	0.0183 (5)	0.0128 (6)	0.0088 (5)
C2A	0.0445 (7)	0.0270 (6)	0.0387 (6)	0.0158 (5)	0.0193 (5)	0.0125 (5)
C3A	0.0430 (6)	0.0248 (5)	0.0367 (6)	0.0148 (5)	0.0146 (5)	0.0089 (4)
C4A	0.0497 (7)	0.0347 (6)	0.0410 (7)	0.0221 (6)	0.0147 (6)	0.0155 (5)
C41A	0.0560 (8)	0.0432 (7)	0.0516 (8)	0.0255 (7)	0.0188 (7)	0.0121 (6)
C42A	0.0821 (14)	0.0934 (15)	0.0926 (15)	0.0474 (12)	0.0511 (12)	0.0238 (12)
C5A	0.0444 (7)	0.0429 (7)	0.0286 (6)	0.0176 (5)	0.0155 (5)	0.0101 (5)
C6A	0.0473 (7)	0.0449 (7)	0.0413 (7)	0.0109 (6)	0.0165 (6)	0.0115 (6)
C7A	0.0527 (9)	0.0662 (10)	0.0514 (9)	0.0051 (8)	0.0209 (7)	0.0149 (7)
C8A	0.0474 (9)	0.0962 (14)	0.0509 (9)	0.0189 (9)	0.0241 (7)	0.0170 (9)
C9A	0.0580 (10)	0.0944 (14)	0.0610 (10)	0.0422 (10)	0.0305 (8)	0.0248 (9)
C10A	0.0572 (9)	0.0604 (9)	0.0570 (9)	0.0320 (7)	0.0294 (7)	0.0228 (7)
C11A	0.0398 (6)	0.0322 (6)	0.0412 (6)	0.0167 (5)	0.0188 (5)	0.0158 (5)
C12A	0.0766 (10)	0.0347 (7)	0.0447 (7)	0.0206 (7)	0.0322 (7)	0.0162 (6)
C13A	0.0808 (11)	0.0509 (8)	0.0451 (8)	0.0290 (8)	0.0331 (8)	0.0176 (6)
C14A	0.0557 (8)	0.0652 (9)	0.0489 (8)	0.0288 (7)	0.0307 (7)	0.0302 (7)
C15A	0.0506 (8)	0.0533 (8)	0.0632 (9)	0.0196 (7)	0.0296 (7)	0.0367 (7)
C16A	0.0434 (7)	0.0346 (6)	0.0525 (8)	0.0132 (5)	0.0200 (6)	0.0199 (6)
O1B	0.0573 (6)	0.0272 (4)	0.0411 (5)	0.0150 (4)	0.0212 (4)	0.0092 (3)
O2B	0.0517 (5)	0.0347 (5)	0.0369 (5)	0.0087 (4)	0.0145 (4)	0.0155 (4)
N1B	0.0423 (5)	0.0299 (5)	0.0317 (5)	0.0109 (4)	0.0142 (4)	0.0084 (4)
N2B	0.0461 (6)	0.0243 (5)	0.0351 (5)	0.0105 (4)	0.0164 (4)	0.0094 (4)
C1B	0.0343 (6)	0.0295 (6)	0.0350 (6)	0.0089 (5)	0.0111 (5)	0.0102 (5)
C2B	0.0365 (6)	0.0286 (6)	0.0335 (6)	0.0114 (5)	0.0139 (5)	0.0083 (4)
C3B	0.0401 (6)	0.0242 (5)	0.0344 (6)	0.0111 (5)	0.0167 (5)	0.0093 (4)
C4B	0.0565 (8)	0.0412 (7)	0.0344 (6)	0.0142 (6)	0.0202 (6)	0.0079 (5)
C41B	0.0659 (10)	0.0440 (8)	0.0355 (7)	0.0108 (7)	0.0134 (7)	0.0062 (6)
C42B	0.0675 (12)	0.0817 (14)	0.0557 (11)	−0.0076 (11)	0.0054 (9)	0.0042 (9)
C5B	0.0357 (6)	0.0294 (5)	0.0341 (6)	0.0096 (5)	0.0181 (5)	0.0115 (4)
C6B	0.0458 (7)	0.0348 (6)	0.0397 (6)	0.0167 (5)	0.0212 (5)	0.0126 (5)
C7B	0.0504 (8)	0.0481 (8)	0.0487 (8)	0.0255 (6)	0.0217 (6)	0.0212 (6)
C8B	0.0439 (7)	0.0570 (8)	0.0387 (7)	0.0173 (6)	0.0139 (6)	0.0176 (6)
C9B	0.0509 (8)	0.0478 (7)	0.0351 (7)	0.0141 (6)	0.0172 (6)	0.0053 (5)
C10B	0.0432 (7)	0.0381 (6)	0.0384 (6)	0.0157 (5)	0.0186 (5)	0.0082 (5)
C11B	0.0373 (6)	0.0303 (6)	0.0353 (6)	0.0093 (5)	0.0145 (5)	0.0038 (5)
C12B	0.0433 (7)	0.0454 (7)	0.0433 (7)	0.0119 (6)	0.0200 (6)	0.0142 (6)
C13B	0.0489 (8)	0.0583 (9)	0.0491 (8)	0.0026 (7)	0.0253 (7)	0.0089 (7)
C14B	0.0443 (8)	0.0671 (10)	0.0767 (11)	0.0096 (8)	0.0330 (8)	0.0020 (9)
C15B	0.0524 (10)	0.0679 (11)	0.1175 (17)	0.0294 (9)	0.0429 (11)	0.0260 (11)
C16B	0.0499 (8)	0.0485 (8)	0.0838 (11)	0.0233 (7)	0.0332 (8)	0.0257 (8)

### 5,5-Diphenyl-3-(2-propyn-1-yl)imidazolidine-2,4-dione Geometric parameters (Å, º)

**Table:**

O1A—C2A	1.2098 (15)	O1B—C2B	1.2062 (15)
O2A—C1A	1.2240 (16)	O2B—C1B	1.2192 (15)
N1A—C2A	1.3638 (16)	N1B—C2B	1.3690 (16)
N1A—C1A	1.3962 (15)	N1B—C1B	1.4006 (15)
N1A—C4A	1.4581 (16)	N1B—C4B	1.4550 (16)
N2A—C1A	1.3394 (18)	N2B—C1B	1.3434 (16)
N2A—C3A	1.4624 (15)	N2B—C3B	1.4647 (14)
N2A—H2A	0.88 (2)	N2B—H2B	0.885 (18)
C2A—C3A	1.5438 (16)	C2B—C3B	1.5427 (16)
C3A—C11A	1.5311 (18)	C3B—C5B	1.5276 (16)
C3A—C5A	1.5332 (19)	C3B—C11B	1.5365 (17)
C4A—C41A	1.456 (2)	C4B—C41B	1.454 (2)
C4A—H4AA	0.9900	C4B—H4BA	0.9900
C4A—H4AB	0.9900	C4B—H4BB	0.9900
C41A—C42A	1.171 (3)	C41B—C42B	1.175 (3)
C42A—H42A	0.9500	C42B—H42B	0.9500
C5A—C6A	1.385 (2)	C5B—C6B	1.3877 (18)
C5A—C10A	1.390 (2)	C5B—C10B	1.3941 (17)
C6A—C7A	1.390 (2)	C6B—C7B	1.3936 (19)
C6A—H6A	0.9500	C6B—H6B	0.9500
C7A—C8A	1.371 (3)	C7B—C8B	1.379 (2)
C7A—H7A	0.9500	C7B—H7B	0.9500
C8A—C9A	1.370 (3)	C8B—C9B	1.383 (2)
C8A—H8A	0.9500	C8B—H8B	0.9500
C9A—C10A	1.387 (2)	C9B—C10B	1.3851 (19)
C9A—H9A	0.9500	C9B—H9B	0.9500
C10A—H10A	0.9500	C10B—H10B	0.9500
C11A—C16A	1.3861 (17)	C11B—C16B	1.384 (2)
C11A—C12A	1.3896 (19)	C11B—C12B	1.3886 (18)
C12A—C13A	1.381 (2)	C12B—C13B	1.389 (2)
C12A—H12A	0.9500	C12B—H12B	0.9500
C13A—C14A	1.378 (2)	C13B—C14B	1.378 (3)
C13A—H13A	0.9500	C13B—H13B	0.9500
C14A—C15A	1.371 (2)	C14B—C15B	1.380 (3)
C14A—H14A	0.9500	C14B—H14B	0.9500
C15A—C16A	1.391 (2)	C15B—C16B	1.382 (2)
C15A—H15A	0.9500	C15B—H15B	0.9500
C16A—H16A	0.9500	C16B—H16B	0.9500
			
C2A—N1A—C1A	112.05 (10)	C2B—N1B—C1B	112.22 (10)
C2A—N1A—C4A	124.18 (10)	C2B—N1B—C4B	124.32 (10)
C1A—N1A—C4A	123.76 (11)	C1B—N1B—C4B	123.46 (10)
C1A—N2A—C3A	113.62 (10)	C1B—N2B—C3B	113.42 (10)
C1A—N2A—H2A	120.4 (13)	C1B—N2B—H2B	121.1 (11)
C3A—N2A—H2A	126.0 (13)	C3B—N2B—H2B	124.3 (11)
O2A—C1A—N2A	128.83 (12)	O2B—C1B—N2B	129.21 (11)
O2A—C1A—N1A	123.93 (12)	O2B—C1B—N1B	123.82 (12)
N2A—C1A—N1A	107.24 (11)	N2B—C1B—N1B	106.96 (10)
O1A—C2A—N1A	125.27 (11)	O1B—C2B—N1B	125.31 (11)
O1A—C2A—C3A	127.78 (11)	O1B—C2B—C3B	128.21 (11)
N1A—C2A—C3A	106.95 (9)	N1B—C2B—C3B	106.44 (9)
N2A—C3A—C11A	113.17 (10)	N2B—C3B—C5B	110.86 (9)
N2A—C3A—C5A	111.85 (11)	N2B—C3B—C11B	112.38 (10)
C11A—C3A—C5A	108.63 (10)	C5B—C3B—C11B	110.51 (9)
N2A—C3A—C2A	100.12 (9)	N2B—C3B—C2B	100.51 (9)
C11A—C3A—C2A	110.22 (10)	C5B—C3B—C2B	115.22 (10)
C5A—C3A—C2A	112.73 (10)	C11B—C3B—C2B	107.02 (9)
C41A—C4A—N1A	112.40 (11)	C41B—C4B—N1B	111.36 (13)
C41A—C4A—H4AA	109.1	C41B—C4B—H4BA	109.4
N1A—C4A—H4AA	109.1	N1B—C4B—H4BA	109.4
C41A—C4A—H4AB	109.1	C41B—C4B—H4BB	109.4
N1A—C4A—H4AB	109.1	N1B—C4B—H4BB	109.4
H4AA—C4A—H4AB	107.9	H4BA—C4B—H4BB	108.0
C42A—C41A—C4A	179.38 (19)	C42B—C41B—C4B	178.6 (2)
C41A—C42A—H42A	180.0	C41B—C42B—H42B	180.0
C6A—C5A—C10A	118.73 (14)	C6B—C5B—C10B	119.12 (11)
C6A—C5A—C3A	122.97 (12)	C6B—C5B—C3B	123.80 (11)
C10A—C5A—C3A	118.21 (12)	C10B—C5B—C3B	117.08 (11)
C5A—C6A—C7A	120.32 (15)	C5B—C6B—C7B	120.06 (12)
C5A—C6A—H6A	119.8	C5B—C6B—H6B	120.0
C7A—C6A—H6A	119.8	C7B—C6B—H6B	120.0
C8A—C7A—C6A	120.52 (17)	C8B—C7B—C6B	120.41 (13)
C8A—C7A—H7A	119.7	C8B—C7B—H7B	119.8
C6A—C7A—H7A	119.7	C6B—C7B—H7B	119.8
C9A—C8A—C7A	119.53 (16)	C7B—C8B—C9B	119.77 (13)
C9A—C8A—H8A	120.2	C7B—C8B—H8B	120.1
C7A—C8A—H8A	120.2	C9B—C8B—H8B	120.1
C8A—C9A—C10A	120.74 (17)	C8B—C9B—C10B	120.18 (13)
C8A—C9A—H9A	119.6	C8B—C9B—H9B	119.9
C10A—C9A—H9A	119.6	C10B—C9B—H9B	119.9
C9A—C10A—C5A	120.14 (16)	C9B—C10B—C5B	120.45 (12)
C9A—C10A—H10A	119.9	C9B—C10B—H10B	119.8
C5A—C10A—H10A	119.9	C5B—C10B—H10B	119.8
C16A—C11A—C12A	119.00 (12)	C16B—C11B—C12B	119.12 (13)
C16A—C11A—C3A	121.92 (12)	C16B—C11B—C3B	121.29 (12)
C12A—C11A—C3A	118.98 (11)	C12B—C11B—C3B	119.56 (11)
C13A—C12A—C11A	120.61 (13)	C11B—C12B—C13B	120.12 (14)
C13A—C12A—H12A	119.7	C11B—C12B—H12B	119.9
C11A—C12A—H12A	119.7	C13B—C12B—H12B	119.9
C14A—C13A—C12A	120.14 (15)	C14B—C13B—C12B	120.38 (15)
C14A—C13A—H13A	119.9	C14B—C13B—H13B	119.8
C12A—C13A—H13A	119.9	C12B—C13B—H13B	119.8
C15A—C14A—C13A	119.74 (14)	C13B—C14B—C15B	119.45 (15)
C15A—C14A—H14A	120.1	C13B—C14B—H14B	120.3
C13A—C14A—H14A	120.1	C15B—C14B—H14B	120.3
C14A—C15A—C16A	120.70 (13)	C14B—C15B—C16B	120.52 (17)
C14A—C15A—H15A	119.7	C14B—C15B—H15B	119.7
C16A—C15A—H15A	119.7	C16B—C15B—H15B	119.7
C11A—C16A—C15A	119.80 (13)	C15B—C16B—C11B	120.37 (16)
C11A—C16A—H16A	120.1	C15B—C16B—H16B	119.8
C15A—C16A—H16A	120.1	C11B—C16B—H16B	119.8
			
C3A—N2A—C1A—O2A	−178.94 (16)	C3B—N2B—C1B—O2B	179.76 (13)
C3A—N2A—C1A—N1A	0.59 (17)	C3B—N2B—C1B—N1B	−0.82 (14)
C2A—N1A—C1A—O2A	178.13 (15)	C2B—N1B—C1B—O2B	−175.41 (12)
C4A—N1A—C1A—O2A	−2.1 (2)	C4B—N1B—C1B—O2B	3.8 (2)
C2A—N1A—C1A—N2A	−1.43 (17)	C2B—N1B—C1B—N2B	5.13 (14)
C4A—N1A—C1A—N2A	178.33 (12)	C4B—N1B—C1B—N2B	−175.67 (12)
C1A—N1A—C2A—O1A	−178.06 (13)	C1B—N1B—C2B—O1B	175.24 (12)
C4A—N1A—C2A—O1A	2.2 (2)	C4B—N1B—C2B—O1B	−3.9 (2)
C1A—N1A—C2A—C3A	1.63 (15)	C1B—N1B—C2B—C3B	−7.01 (14)
C4A—N1A—C2A—C3A	−178.13 (12)	C4B—N1B—C2B—C3B	173.80 (12)
C1A—N2A—C3A—C11A	117.61 (13)	C1B—N2B—C3B—C5B	−125.40 (11)
C1A—N2A—C3A—C5A	−119.29 (13)	C1B—N2B—C3B—C11B	110.38 (12)
C1A—N2A—C3A—C2A	0.34 (15)	C1B—N2B—C3B—C2B	−3.09 (13)
O1A—C2A—C3A—N2A	178.52 (13)	O1B—C2B—C3B—N2B	−176.45 (13)
N1A—C2A—C3A—N2A	−1.16 (13)	N1B—C2B—C3B—N2B	5.88 (12)
O1A—C2A—C3A—C11A	59.07 (17)	O1B—C2B—C3B—C5B	−57.26 (17)
N1A—C2A—C3A—C11A	−120.61 (11)	N1B—C2B—C3B—C5B	125.07 (11)
O1A—C2A—C3A—C5A	−62.49 (17)	O1B—C2B—C3B—C11B	66.05 (16)
N1A—C2A—C3A—C5A	117.83 (11)	N1B—C2B—C3B—C11B	−111.62 (11)
C2A—N1A—C4A—C41A	−80.14 (16)	C2B—N1B—C4B—C41B	−68.42 (17)
C1A—N1A—C4A—C41A	100.12 (16)	C1B—N1B—C4B—C41B	112.48 (14)
N2A—C3A—C5A—C6A	132.60 (12)	N2B—C3B—C5B—C6B	117.89 (12)
C11A—C3A—C5A—C6A	−101.77 (13)	C11B—C3B—C5B—C6B	−116.84 (12)
C2A—C3A—C5A—C6A	20.69 (16)	C2B—C3B—C5B—C6B	4.61 (16)
N2A—C3A—C5A—C10A	−50.88 (15)	N2B—C3B—C5B—C10B	−62.07 (14)
C11A—C3A—C5A—C10A	74.75 (14)	C11B—C3B—C5B—C10B	63.20 (13)
C2A—C3A—C5A—C10A	−162.79 (12)	C2B—C3B—C5B—C10B	−175.35 (10)
C10A—C5A—C6A—C7A	1.4 (2)	C10B—C5B—C6B—C7B	0.08 (19)
C3A—C5A—C6A—C7A	177.91 (13)	C3B—C5B—C6B—C7B	−179.87 (12)
C5A—C6A—C7A—C8A	−1.7 (2)	C5B—C6B—C7B—C8B	−0.5 (2)
C6A—C7A—C8A—C9A	1.0 (3)	C6B—C7B—C8B—C9B	0.7 (2)
C7A—C8A—C9A—C10A	0.0 (3)	C7B—C8B—C9B—C10B	−0.4 (2)
C8A—C9A—C10A—C5A	−0.3 (3)	C8B—C9B—C10B—C5B	−0.1 (2)
C6A—C5A—C10A—C9A	−0.4 (2)	C6B—C5B—C10B—C9B	0.22 (19)
C3A—C5A—C10A—C9A	−177.07 (14)	C3B—C5B—C10B—C9B	−179.82 (12)
N2A—C3A—C11A—C16A	10.63 (17)	N2B—C3B—C11B—C16B	−13.23 (17)
C5A—C3A—C11A—C16A	−114.22 (13)	C5B—C3B—C11B—C16B	−137.64 (13)
C2A—C3A—C11A—C16A	121.81 (13)	C2B—C3B—C11B—C16B	96.18 (14)
N2A—C3A—C11A—C12A	−172.91 (12)	N2B—C3B—C11B—C12B	168.84 (11)
C5A—C3A—C11A—C12A	62.23 (15)	C5B—C3B—C11B—C12B	44.43 (15)
C2A—C3A—C11A—C12A	−61.73 (16)	C2B—C3B—C11B—C12B	−81.75 (13)
C16A—C11A—C12A—C13A	−0.7 (2)	C16B—C11B—C12B—C13B	1.4 (2)
C3A—C11A—C12A—C13A	−177.27 (14)	C3B—C11B—C12B—C13B	179.41 (12)
C11A—C12A—C13A—C14A	0.3 (3)	C11B—C12B—C13B—C14B	0.4 (2)
C12A—C13A—C14A—C15A	0.2 (3)	C12B—C13B—C14B—C15B	−1.8 (3)
C13A—C14A—C15A—C16A	−0.3 (2)	C13B—C14B—C15B—C16B	1.3 (3)
C12A—C11A—C16A—C15A	0.6 (2)	C14B—C15B—C16B—C11B	0.6 (3)
C3A—C11A—C16A—C15A	177.08 (12)	C12B—C11B—C16B—C15B	−1.9 (2)
C14A—C15A—C16A—C11A	−0.1 (2)	C3B—C11B—C16B—C15B	−179.86 (16)

### 5,5-Diphenyl-3-(2-propyn-1-yl)imidazolidine-2,4-dione Hydrogen-bond geometry (Å, º)

*Cg*2 and *Cg*3 are the centroids of the C5*A*–C10*A* and C11*A*–C16*A* benzene rings, respectively.

**Table:**

*D*—H···*A*	*D*—H	H···*A*	*D*···*A*	*D*—H···*A*
N2*A*—H2*A*···O2*A*^i^	0.88 (2)	1.99 (2)	2.855 (2)	167 (2)
N2*B*—H2*B**B*···O2*B*^ii^	0.89 (2)	1.99 (2)	2.847 (1)	163 (2)
C4*A*—H4*AA*···O1*A*^iii^	0.99	2.31	3.253 (2)	160
C15*A*—H15*A*···O1*B*	0.95	2.44	3.332 (2)	156
C8*B*—H8*B*···C*g*2^i^	0.95	2.88	3.713 (2)	147
C15*B*—H15*B*···C*g*3^iv^	0.95	2.87	3.742 (3)	153

Symmetry codes: (i) −*x*+1, −*y*+1, −*z*; (ii) −*x*+1, −*y*, −*z*+1; (iii) −*x*+1, −*y*+2, −*z*; (iv) −*x*+2, −*y*+1, −*z*+1.
